# Nickel-Graphene Nanoplatelet Deposited on Carbon Fiber as Binder-Free Electrode for Electrochemical Supercapacitor Application

**DOI:** 10.3390/polym12081666

**Published:** 2020-07-27

**Authors:** Hemraj M. Yadav, Narayan Chandra Deb Nath, Jeonghun Kim, S. K. Shinde, Sivalingam Ramesh, Faruk Hossain, Olaniyan Ibukun, Jae-Joon Lee

**Affiliations:** 1 Research Center for Photoenergy Harvesting & Conversion Technology (phct), Department of Energy and Materials Engineering, Dongguk University, Seoul 04620, Korea; hemrajy@dongguk.edu (H.M.Y.); rahul101db@gmail.com (N.C.D.N.); faruk17july@gmail.com (F.H.); olaniyanibukun0@gmail.com (O.I.); 2 Department of Chemistry, Kookmin University, 77 Jeongneung-ro, Seongbuk-gu, Seoul 02707, Korea; jeonghunkim@kookmin.ac.kr; 3 Department of Biological and Environmental Science, Dongguk University-Ilsan, Biomedical Campus, Goyang-si, Gyeonggi-do 10326, Korea; shindesurendra9@gmail.com; 4 Department of Mechanical, Robotics and Energy Engineering, Dongguk University, Seoul 04620, Korea; sivaramesh_74@yahoo.co.in

**Keywords:** graphene, nickel, electrochemical synthesis, electrochemical capacitor

## Abstract

A binder-free process for the electrode preparation for supercapacitor application was suggested by drop casting graphene nanoplatelets on a carbon fiber (GnP@CF) followed by electrodeposition of Ni nanoparticles (NPs). The microstructure of the electrode showed that Ni was homogeneously distributed over the surface of the GnP@CF. XRD analysis confirmed the cubic structure of metallic Ni NPs. The Ni-GnP@CF electrode showed excellent pseudocapacitive behavior in alkaline solution by exhibiting a specific capacitance of 480 F/g at 1.0 A/g, while it was 375 F/g for Ni@CF. The low value of series resistance of Ni-GnP@CF (1 Ω) was attributed to the high capacitance. The enhanced capacitance of the electrode could be correlated to the highly nanoporous structure of the composite material, synergetic effect of the electrical double layer charge-storage properties of graphene, and the pseudocapacitive nature of Ni NPs.

## 1. Introduction

The supercapacitor (SC) is well-known and promising for energy storage devices because of its high power density, extensive life cycles, and easy fabrication and maintenance [1,2,3,4]. SCs are mainly classified according to the charge storage mechanism; electrochemical double layer capacitors (EDLCs) stores the energy by non-Faradic process by the accumulation of the charges at the electrode–electrolyte interface, while it is done by Faradic process for the pseudo capacitor (a reduction-oxidation based capacitor) as in batteries [5,6].

Generally, carbonaceous materials, such as activated carbon, single and multiwalled carbon nanotubes, and graphene, are used as electrode materials in double-layer capacitors [7,8,9,10,11]. Graphene is a carbonaceous material with numerous exclusive features, such as 2D plane structure united with one-atom thickness and extraordinary physiochemical properties. Graphene has inspired exploration in energy material applications, owing to its high surface area, mechanical strength, and electrical conductivity [1,8,12,13]. The overall performance of SC based on carbon materials is quite low due to the limited charge-storage capacity.

Meanwhile, pseudocapacitive materials, such as transition metals and metal oxides, and functionalized or doped graphene, have been studied for use in SCs owing to their larger specific capacitance and higher energy density compared to pristine carbon-based materials [14]. Among various metals and metal oxides, earth-abundant and cost-effective nickel-based nanomaterials have been used widely for energy storage applications due to the high theoretical specific capacitance, electrical conductivity, specific surface area, and variable oxidation state [15,16,17,18,19,20,21,22].

Graphene-nickel based nanostructures, such as graphene-Ni(OH)_2_ and graphene-NiO, were studied in depth for hybrid SC applications [16]. Further, these nanostructures have several potential applications in the field of catalysis, nanoelectronics, sensors, photovoltaics, energy, and healthcare [23]. However, very few reports are available on the graphene-Ni NPs composites for electrochemical SCs applications [24,25]. Ni NPs based electrocatalysts are the most promising, due to its remarkable reversible redox reaction of Ni(OH)_2_/NiOOH in alkaline solution. The combination of Ni with highly conductive graphene could boost their electrochemical performance by enhancing the electron transfer between active materials [26,27]. However, these composites were generally prepared by a mechanical mixing process, and it often resulted in a poor adhesion of active materials on the electrode surface. In addition, the post-treatment of the catalyst requires the use of non-conducting polymer binders, which reduces the electrical conductivity of the electrode and it induces the decrease of electrocatalytic activity eventually. It is well known that graphene has relatively poor adherence to substrate, and therefore, it is crucial to develop alternative techniques to increase the stability of graphene and its composites without scarifying the electrical conductivity and electrocatalytic activity.

In the present study, we demonstrated a binder-free process to fabricate Ni-GnP@CF electrode by a simple drop coating of GnP on the CF followed by electrochemical deposition of Ni. The Ni-GnP@CF electrode showed superior electrochemical capacitance of 480 F/g, which was almost two times higher compared to that of the previous reports with nickel-graphene composite electrodes [24,25]. It could be attributed to the highly nanoporous structure, fast reversible redox reaction at Ni surface, strong interconnection between composite material, and synergetic effect of GnP and Ni NPs in the composite.

## 2. Materials and Methods

### 2.1. Materials and Reagents

Nickel (II) sulfate hexahydrate (Ni_2_SO_4_(H_2_O)_6_) and boric acid (BH_3_O_3_) were purchased from Sigma Aldrich, Seoul, Korea. Sodium sulfate (Na_2_SO_4_) and potassium hydroxide were procured from Daejung chemicals, Gyeonggi-do, Korea. Graphene nanoplatelets (GnP) were purchased from XG science, Inc. (Lansing, MI, USA). Graphene nanoplatelets xGnP^®^ 750 grade C had a surface area of 750 m^2^/g. Carbon fiber sheets were obtained from NARA Cell-Tech Corporation, Seoul, Korea.

### 2.2. Apparatus

Electrochemical measurements were performed with an electrochemical analyzer instrument model versa stat 3 using a CF as working electrode, platinum metal foil as counter electrode, and an Ag/AgCl (Sat. KCl) electrode as a reference electrode. Cyclic voltammetry (CV), galvanostatic discharge-charge (GCD) and electrochemical impedance spectroscopy (EIS) were measured using 5 M KOH aqueous solution at room temperature. The specific capacitance value ($C_{s}$) of Ni@CF for GCD study was estimated using the equation $C_{s}=I\;\times \;∆t/m∆v$, where *I* is the applied current density of charge/discharge, *t* is the time of discharge, ∆v is potential different, and m is the mass of active materials on the CF electrode. The active mass was about 0.0008 and 0.0011 g for Ni@CF and Ni-GnP@CF, respectively. The prepared electrodes were characterized by X-ray diffractometer with Cu Kα (λ = 0.15406 nm). The average crystallite size was obtained from the (1 1 1) reflection of nickel at 44.46° using the Debye–Scherer formula: D=(0.94λ)/(β × Cosθ), where λ is the wavelength of incident X-rays, β is the full width at half maximum, and θ is the angle obtained from 2θ value of the most intense peak of Ni. The morphology of the electrode was examined by a field emission scanning electron microscope (JSM 7100 JEOL Ltd., Tokyo, Japan) and transmission electron microscopy (JEM-3010 (JEOL Ltd., Tokyo, Japan). The Ni-GnP@CF electrode was scratched and transferred to the copper grid for TEM analysis. Raman spectra were measured on a Micro Raman spectrometer (Nanofinder 30, Tokyo Instruments Inc., Tokyo, Japan) using a laser operating at a wavelength of approximately 532 nm. The X-ray photoelectron spectroscopy (XPS) measurements were performed using a PHI 5000 versa probe spectrometer with monochromatic Al Kα radiation (hν = 1253.6 eV, Kanagawa, Japan).

### 2.3. Fabrication of Ni@CF and Ni-GnP@CF Electrode

**Figure 1:**
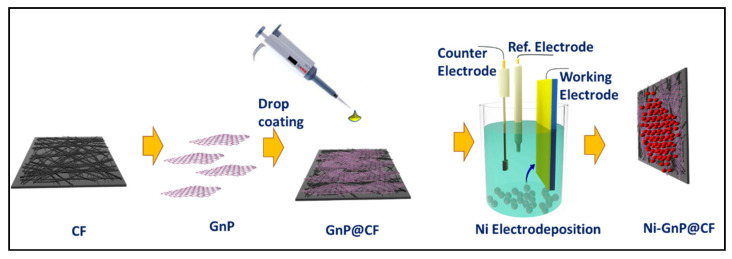
Schematic for fabrication of Ni-GnP@CF.

A simple drop coating technique was applied for the preparation of GnP@CF. For this, CF was drop coated with commercially available graphene XG-750C dispersed in isopropanol (100 μL, 1 mg/mL) and dried in air, then at 70 °C for 30 min in an electric oven. The electrochemical deposition of Ni NPs on the CF and GnP@CF was performed in an electrolyte, including 0.05 M Ni_2_SO_4_(H_2_O)_6_, 0.05 M Na_2_SO_4_, and 0.05 M BH_3_O_3_. A potential of −1.0 V vs. Ag/AgCl (Sat. KCl) was applied to a working CF electrode for 1500 s. Nitrogen gas was bubbled in the solution for 30 min prior to the deposition of Ni on CF. Then, the electrode was washed with distilled water and dried at 80 °C for 30 min in an electric oven. The schematic diagram of the experiment is displayed in Figure 1.

## 3. Results and Discussion

**Figure 2:**
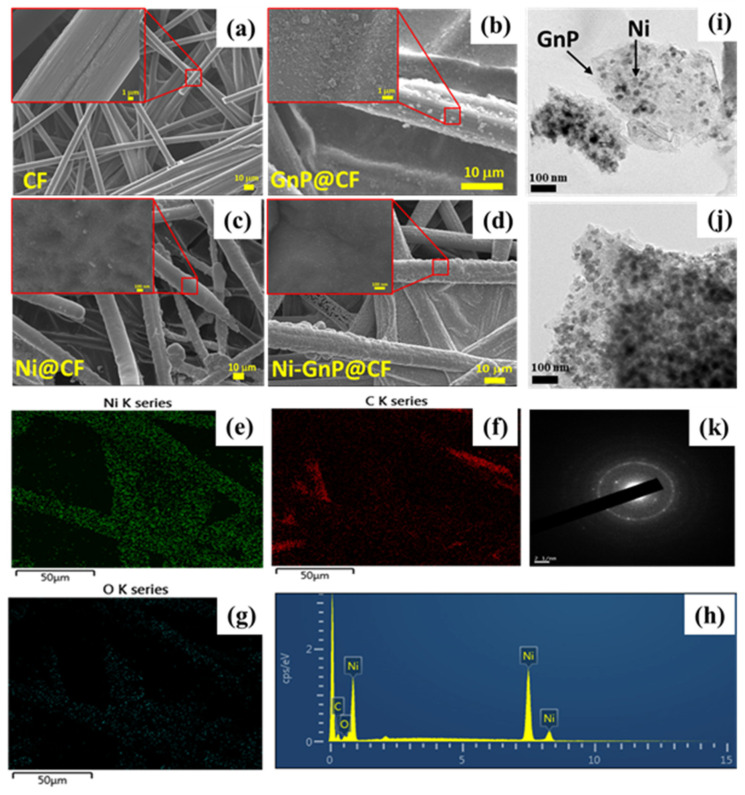
FESEM image of CF (**a**), GnP@CF (**b**), Ni@CF (**c**) Ni-GnP@CF (**d**); EDS mapping (**e**–**g**), EDS spectrum (**h**), TEM images (**i**,**j**), and SEAD pattern (**k**) of Ni-GnP@CF.

Figure 2 shows FESEM images of CF, GnP@CF, Ni@CF, and Ni-GnP@CF electrode. The surface of pristine CF was smooth, as shown in Figure 2a. Figure 2b indicates the uniform anchoring of GnP over the CF. Figure 2c and Figure S1 shows EFSEM images of Ni NPs deposited on CF by electrochemical method with different magnifications. FESEM shows well decorated spheres like the structure of Ni NPs on the CF and GnP@CF electrode (Figure 2c–d). Energy-dispersive X-ray spectroscopy (EDS) mappings were carried to additionally examine the nature of the Ni@CF and Ni-GnP@CF electrodes. Figure 2e–h displays the elemental mapping and EDS spectra of nickel, carbon, and oxygen of Ni-GnP@CF electrodes, respectively. Similarly, the elemental mapping and EDS spectra of Ni@CF is shown in Figure S1. These elemental maps demonstrate that the graphene was well-covered by the Ni NPs, which is also consistent with the FESEM and TEM results [28].

The TEM images and selected area electron diffraction (SAED) pattern of the Ni-GnP@CF sample is depicted in Figure 2i–k, respectively. The Ni NPs are uniform in size of about 25 nm with a globular shape. These NPs were well decorated on the surface of the graphene sheet. The SAED pattern of the Ni-GnP@CF sample was composed of bright dots as well as rings (Figure 2k). The diffraction ring pattern confirms the polycrystalline nature of the Ni NPs.

**Figure 3:**
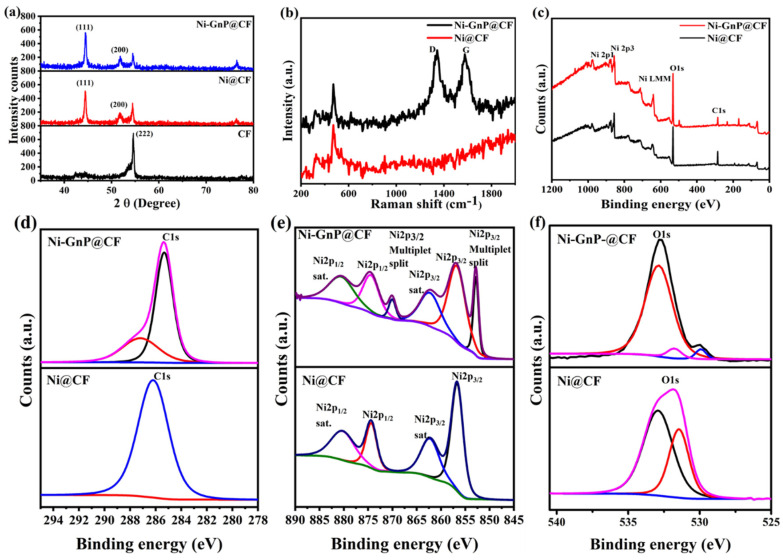
(**a**) XRD patterns of CF, Ni@CF, and Ni-GnP@CF, (**b**) Raman spectra of Ni@CF and Ni-GnP@CF, (**c**) XPS survey spectra, (**d**) C1s, (**e**) core level Ni2p, and (**f**) core level O1s of Ni@CF and Ni-GnP@CF.

Figure 3a and Figure S2 shows XRD patterns of CF, Ni@CF, and Ni-GnP@CF. The XRD peaks of Ni-GnP@CF were indexed with cubic metallic Ni [JCPDS 01-070-1849] and graphitic carbon from CF. The XRD peaks at ≈44.46°, 51.82°, and 76.54° correlated with the characteristic planes, such as (1 1 1), (2 0 0), and (2 2 0), respectively, of cubic metallic nickel [29]. The most intense peak of graphitic carbon from CF at about 26.38° was interestingly suppressed with the deposition of Ni on CF, and further, intensity decreased for Ni-GnP@CF (Figure S2). The XRD peak at 26.38° and 54.52° matches with the standard XRD peaks of graphite (JCPDS 01-075-2078); its intensity was gradually suppressed with the incorporation of Ni NPs. The decrease in the intensity of the XRD peak for graphitic carbon with Ni loading demonstrates the distribution of Ni on the surface of the Supporting Material. The XRD peaks for oxides of nickel with diverse phases were not observed [29]. The average crystallite size was estimated by the Debye–Scherer formula [30]. The average crystallite sizes of Ni NPs were about 38 and 32 nm for Ni@CF and Ni-GnP@CF, respectively. The existence of GnP might be favorable for the formation of small crystallites of Ni on the Ni-GnP@CF by increasing electrical conductivity of the electrode and providing higher surface area.

Figure 3b shows the Raman spectra of Ni@CF and Ni-GnP@CF. The peak located at 472.9 cm^−1^ was detected for Ni@CF and Ni-GnP@CF, indicating that the Ni was well bonded with the GnP. These results are consistent with previous reports [31,32]. The Raman spectrum of Ni-GnP@CF exhibits the combination of the characteristic peaks of Ni and GnP. The D band (1342.6 cm^−1^) is attributed to the edge or in-plane sp^3^ defects, as well as disordered carbon; while the G band (1575.6 cm^−1^) is attributed to the in-plane vibration of ordered sp^2^-bonded carbon atoms [33]. The intensity ratio, I_D_/I_G_, has a high value of about 1.04, which reveals the high quality of GnP [7].

The chemical compositions of the Ni@CF and Ni-GnP@CF were studied using XPS in the range of 0–1200 eV. The survey spectra of both samples are shown in Figure 3c. The C1s spectrum of Ni@CF shows only one peak at 286.19 eV, confirming the bond between Ni and CF; while the two peaks observed for Ni-GnP@CF at 285.33 and 287.21 eV can be assigned to carbon atoms in C–O and C=O, respectively (Figure 3d) [28]. Figure 3e shows the characteristic core level. The two main peaks at 874.35 and 856.66 eV in Ni2p XPS spectra were assigned to Ni2p1/2 and Ni2p3/2, respectively, with a spin-energy separation of 17.6 eV for metallic Ni on Ni@CF. Furthermore, satellite peaks (Ni2p1/2, satellite: 880.24 eV; Ni2p3/2, satellite: 862.25 eV) were also observed, which arise due to the Ni(OH)2 phase. [34,35]. Similarly, for Ni-GnP@CF, the peaks at 874.41 and 856.74 eV were assigned to Ni2p1/2 and Ni2p3/2, respectively. Further, the characteristic core level peaks of nickel Ni2p3/2 and Ni2p1/2 at 852.81 and 870.04 eV, respectively, corresponds to Ni0 [36]. The satellite peaks were generally located at ≈6 eV, followed by the main 2p peaks for metallic nickel. The absence of any doublet peak around 854 eV indicates the absence of nickel oxide (NiO) [37]. Here, two O1s components for Ni@CF (Figure 3f) at 531.42 and 532.84 eV could be assigned to the existence of surface hydroxyl groups and chemically or physically adsorbed water, respectively [38]. Similarly, for Ni-GnP@CF, the O1s core level peaks at 529.76, 531.55, and 532.91 eV correspond to the C=O group, surface hydroxyl groups, and chemically or physically adsorbed water, respectively [7,17,39].

### Electrochemical Behavior

**Figure 4:**
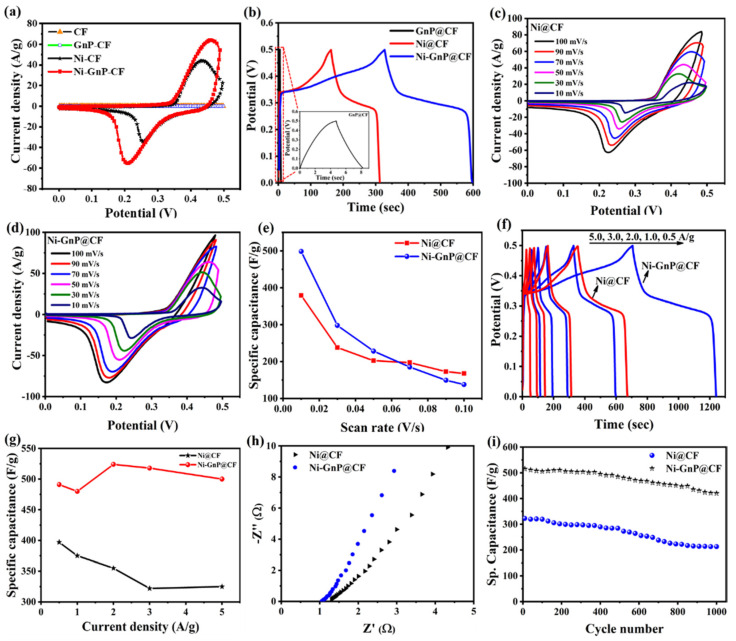
(**a**) Cyclic voltammogram of CF, GnP@CF, Ni@CF, and Ni-GnP@CF at 50 mV/s in 5 M KOH; (**b**) GCD curve of GnP@CF, Ni@CF, and Ni-GnP@CF at 1.0 A/g in 5 M KOH, inset magnified image of GnP@CF; (**c**) CV of Ni@CF; (**d**) CV of Ni@GCCF in 5 M KOH; (**e**) specific capacitance vs. scan rate for Ni@CF and Ni-GnP@CF; (**f**) GCD for Ni@CF and Ni-GnP@CF; (**g**) specific capacitance vs. current density for Ni@CF and Ni-GnP@CF; (**h**) Nyquist plots of Ni@CF and Ni-GnP@CF electrodes; and (**i**) cycling stability of Ni@CF and Ni-GnP@CF at 5.0 A/g.

The CV of CF, GnP@CF, Ni@CF, and Ni-GnP@CF were measured at 50 mV/s in 5 M KOH to investigate electrochemical behavior of each material (Figure 4a). The Ni@CF and Ni-GnP@CF showed very strong reversible redox peaks compared with CF and GnP@CF due to the three-dimensional deposition of Ni NPs and faradaic transformations of Ni^2+^/Ni^3+^. CV of the CF and GnP@CF showed a rectangular shape without any redox peak, which is characteristics of electrical double layer capacitance (EDLC) behavior of GnP. The shape of CV for Ni@CF and Ni-GnP@CF are obviously different from those of CF and GnP@CF due to the strong reduction and oxidation reactions.

Figure 4c–d showed the CV curves of the Ni@CF and Ni-GnP@CF electrodes at different scan rates, which demonstrate the behavior of current density values with scan rates. The peak potential of Ni-GnP@CF shifts negatively compared to Ni@CF, revealing enhancement in the electrochemical kinetic Ni NPs in presence of GnP. Additionally, the plot for peak current density vs. square root of scan rates are shown in Figure S3. The cathodic and anodic peak currents are linearly proportional to the square root of the scan rates, which indicates the kinetics of the electrochemical reaction of the electrodes [40].

The nature of redox peaks in the CV curves and increase in redox peaks with increasing scan rate, reveals that the specific capacitance of the Ni-GnP@CF electrode was mainly attributed to pseudocapacitive behavior, depending on the reversible faradaic transitions of Ni^2+^/Ni^3+^ [28,41]. The anodic peak at about 0.45 V (vs. Ag/AgCl) was due to the oxidation of a few monolayers of surface Ni(OH)_2_ to NiOOH, whereas the cathodic peak at 0.18 V (vs. Ag/AgCl) results from the reverse reduction process [28,42]. This indicates the pseudocapacitance performance of the prepared electrode [26]. For the Ni@CF electrode, faradaic reactions at the surface are represented as follows:

It is well known that the area under CV was related to the capacitance of material [26]. The area under CV of Ni-GnP@CF was larger than that of Ni@CF, confirming that Ni-GnP@CF had higher capacitance. The specific capacitance values of the Ni@CF and Ni-GnP@CF from CV are depicted in the Figure 4e. The maximum specific capacitance of Ni@CF and Ni-GnP@CF was 379 and 498 F/g for the scan rate of 10 mV/s, respectively.

(1) $$\mathrm{Ni}{\left(\mathrm{OH}\right)}_{2}+{\mathrm{OH}}^{-}↔\mathrm{NiOOH}+{\;H}_{2}O+e^{-}$$

The GCD for GnP@CF, Ni@CF, and Ni-GnP@CF at 1.0 A/g in 5 M KOH is shown in Figure 4b. The GCD of Ni@CF and Ni-GnP@CF exhibit a plateau at 0.25–0.35 V, which is characteristic of pseudo capacitive nature. This demonstrated that the Ni-GnP@CF electrode had a high capability for SC application. The enhanced electrochemical performance can be attributed to the interaction between Ni and GnP because of EDLC from GnP and the pseudo capacitance arising from Ni sites [25]. It is well-known that the behavior of nickel-based materials in an alkaline condition has been discussed in previous reports [15,41]. Graphene with high conductivity and high surface area stores the charge via the formation of the Helmholtz layer, while the Ni NPs exhibits a fast-faradaic reaction during charge-storage. Therefore, the composite materials showed significantly higher capacitive performance, compared with the individual components.

The electrochemical SC properties of Ni@CF and Ni-GnP@CF electrodes were studied by GCD measurements with 5 M KOH aqueous solution in the potential range of 0–0.5 V vs. Ag/AgCl electrode. Figure 4f shows the GCD plots of Ni@CF and Ni-GnP@CF at 0.5, 1.0, 2.0, 3.0, and 5.0 A/g. The charging time and discharging time are nearly identical, suggesting a high reversibility of the faradaic reaction occurring on the Ni surface [15]. The charging time and discharging time of Ni-GnP@CF electrode for each cycle was almost double than that of the Ni@CF electrode, showing that the porous nature of the material and strong interconnection with support can improve the capacitance performance of Ni-GnP@CF electrode [15].

The estimated specific capacitance of the Ni@CF was 397, 375, 355, 322, 325 F/g, while for Ni-GnP@CF it was about 491, 480, 524, 518, 500 F/g, at 0.5, 1.0, 2.0, 3.0, and 5.0 A/g, respectively (Figure 4g). The specific capacitance of Ni-GnP@CF increased after the current density of 1 A/g due to the higher ionic penetration in the electrode surface compared to low current rate. The higher capacitance can be attributed to the existence of graphene and its interconnection with Ni without binder, which was confirmed from Raman analysis. The confinement of graphene increased conductivity and allowed dispersion of Ni NPs on its surface. These results demonstrate that Ni-GnP composites can act as a decent electroactive material for SC application, and our results are comparable with previously reported literature (Table 1).

Figure 4h presents the Nyquist plot of the electrodes, measured in the frequency range of 100 kHz to 0.01 Hz, with an AC excitation signal of 4 mV. The almost straight line at small frequency ranges demonstrates lower diffusion resistance of the Ni-GnP@CF than the Ni@CF electrode. This may be due to highly nanoporous structures formed with graphene sheets and Ni NPs, which offers large surface area for the easy diffusion of electrolyte ions [26]. The equivalence series resistance of Ni-GnP@CF and Ni@CF electrodes were 1 and 1.34 Ω, respectively. The lower series resistance of Ni-GnP@CF was attributed to its improved electrochemical capacitance.

The cycling stability was carried out in the potential range of 0–0.5 V for over 1000 cycles (Figure 4i). The Ni-GnP@CF pseudo capacitor displays better cycling stability, retaining 81% after 1000 cycles. The rate of capacitance was increased initially at about 100 cycles and reached at maximum value at about 400 cycles. This is due to the penetration of the electrolyte solution and gradual activation of the active material [26,43]. The relative standard deviation of the specific capacitance for 10 pieces of composite electrodes is about 4.3%. The higher and more stable cycling performance of the Ni-GnP@CF electrode material is related to the nanoprorous nature of the samples, electrochemical properties of the materials, and the synergetic effect of GnP and Ni NPs. The SEM images after cycling test confirms the stability of Ni-GnP@CF (Figure S4). The SEM image of Ni-GnP@CF after use shows surface passivation of the electrode, which might be attributed to the decreased rate of capacitance retention. The incorporation of the graphene matrix improved the structural stability upon cycling, and the electrical properties of Ni were enhanced due to GnP support. Further, synergetic effect from the electrical double layer capacitance of GnP and pseudo capacitance of Ni nanostructures also contributed towards enhanced electrochemical performance of Ni-GnP@CF. This reveals that Ni-GnP@CF electrodes are promising for SC application.

## 4. Conclusions

The Ni-GnP@CF electrode was prepared by facile drop casting of GnP on the CF and the following electrochemical deposition of Ni NPs on it. The structural and morphological properties of the composite reveal that Ni NPs, ≈25 nm size, with cubic crystalline structure and without any trace of nickel oxides are homogeneously distributed on the surface of GnP@CF. The specific capacitance of the Ni-GnP@CF electrode, estimated from the GCD curves, was about 480 F/g at 1.0 A/g. This superior performance with excellent cycling stability can be ascribed to the synergistic effect of the electrical double layer charge-storage properties of graphene, pseudo capacitance nature of nickel, and lower series resistance of the composite.

## Figures and Tables

**Table 1:** Parameters of nickel-graphene based SCs and their electrochemical performance.

Materials	Substrate	Synthesis Method	Capacitance (F/g)	Current Density (A/g)	Electrolyte	Ref.
PANMA/graphene/NiO	GCE	Hydrothermal	549	1.0	1 M H_2_SO_4_	[44]
Graphene/NiO	GCE	Hydrothermal-Precipitation	1328	1.0	2 M KOH	[7]
Graphene/NiO	Ni foam	Hydrothermal	342.9	1.0	6 M KOH	[15]
RGO–NiO	Ni foam	Solvothermal	576	1.0	6 M KOH	[40]
Nio/graphene aerogel	Ni foam	Solvothermal	587.3	1.0	6M KOH	[45]
NiO-graphene	Ni foam	Hydrothermal	617	1.0	5 M NaOH	[46]
NiO-graphene	Ni foam	Electrochemical	745	1.4	3 M KOH	[12]
NiO/RGO	Ni foam	Hydrothermal	96	1.0	6 M KOH	[26]
NiO@graphene	Ni foam	Electrophoretic deposition	1258	5.0	6 M KOH	[41]
Carbon-supported NiO	Ni foil	Precipitation	127	1.0	1 M KOH	[17]
Ni-graphene	Ti foil	Solvothermal-ball milling	275	2.0	1 M KOH	[25]
NiO/RGO	Ti foil	Hydrothermal	590	1.0	1 M KOH	[47]
Ni	CF	Electroless deposition	268	0.2	6 M KOH	[24]
Ni-GnP	CF	Drop coating-Electrochemical	480	1.0	5 M KOH	This work

## Supplementary Materials

The following are available online at https://www.mdpi.com/2073-4360/12/8/1666/s1—Figure S1: SEM images with different magnification (a–c), EDS mapping (d–f), and EDS spectrum of Ni@CF (g); Figure S2: XRD pattern of CF, Ni@CF, and Ni-GnP@CF in the range of 20–80°; Figure S3: Plot for peak current density vs. square root of scan rates; and Figure S4: SEM comparison of both electrodes before and after 1000 cycles.
