# Supplementary material for: Nickel-Graphene Nanoplatelet Deposited on Carbon Fiber as Binder-Free Electrode for Electrochemical Supercapacitor Application
Source: Polymers (Basel). 2020 Jul 27;12(8):1666. doi: 10.3390/polym12081666 (PMC7463841; doi:10.3390/polym12081666)
Supplement: Supplementary file 1 [file polymers-12-01666-s001.pdf]

polymers

MDPI

Supporting information

Nickel-Graphene Nanoplatelet Deposited on Carbon Fiber as Binder-free Electrode for Electrochemical Supercapacitor Application

Hemraj M. Yadav[1], Narayan Chandra Deb Nath[1], Jeonghun Kim[2], S.K. Shinde[3], Sivalingam Ramesh[4], Faruk Hossain[1], Olaniyan Ibukun[1] and Jae-Joon Lee[1]*

[1]Research Center for Photoenergy Harvesting & Conversion Technology (phct), Department of Energy and Materials Engineering, Dongguk University, Seoul, 04620, South Korea.

[2]Department of Chemistry, Kookmin University, 77 Jeongneung-ro, Seongbuk-gu, Seoul 02707, South Korea.

[3]Department of Biological and Environmental Science, Dongguk University-Ilsan, Biomedical Campus, Goyang-si, Gyeonggi-do, 10326, South Korea.

[4]Department of Mechanical, Robotics and Energy Engineering, Dongguk University, Seoul, 04620, South Korea.

* Correspondence: jjlee@dongguk.edu; Tel: 82-10-4659-8255 (c), 82-2-2260-4979 / 8513 (Dep Office.) (Jae-Joon Lee, Ph.D., Professor)

Received: date; Accepted: date; Published: date

polymers

MDPI

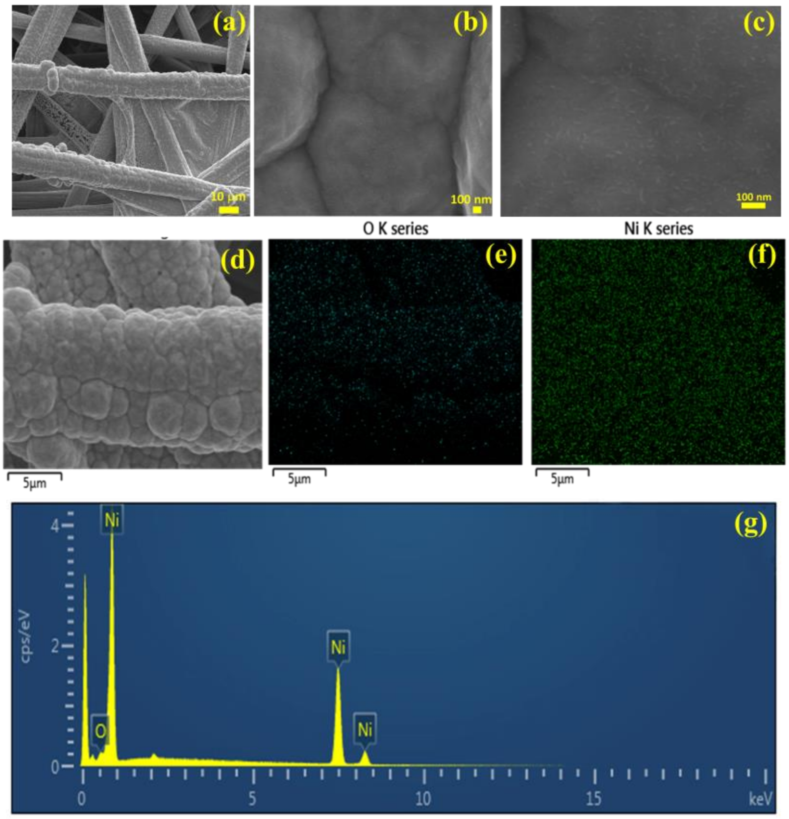

Figure S1. SEM images with different magnification (a-c), EDS mapping (d-f), and EDS spectrum of Ni@CF (g).

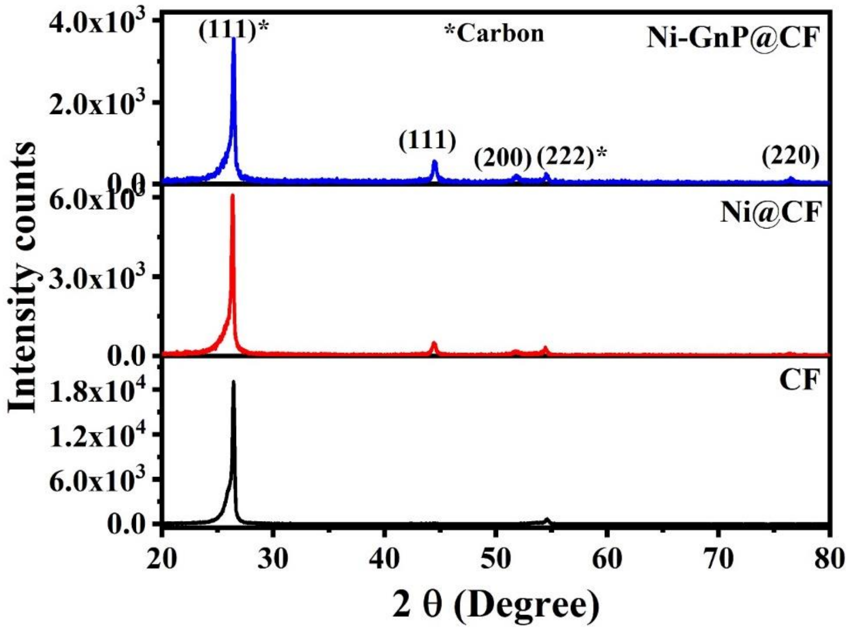

Figure S2. XRD patter of CF, Ni@CF and Ni-GnP@CF in the range of 20-80°.

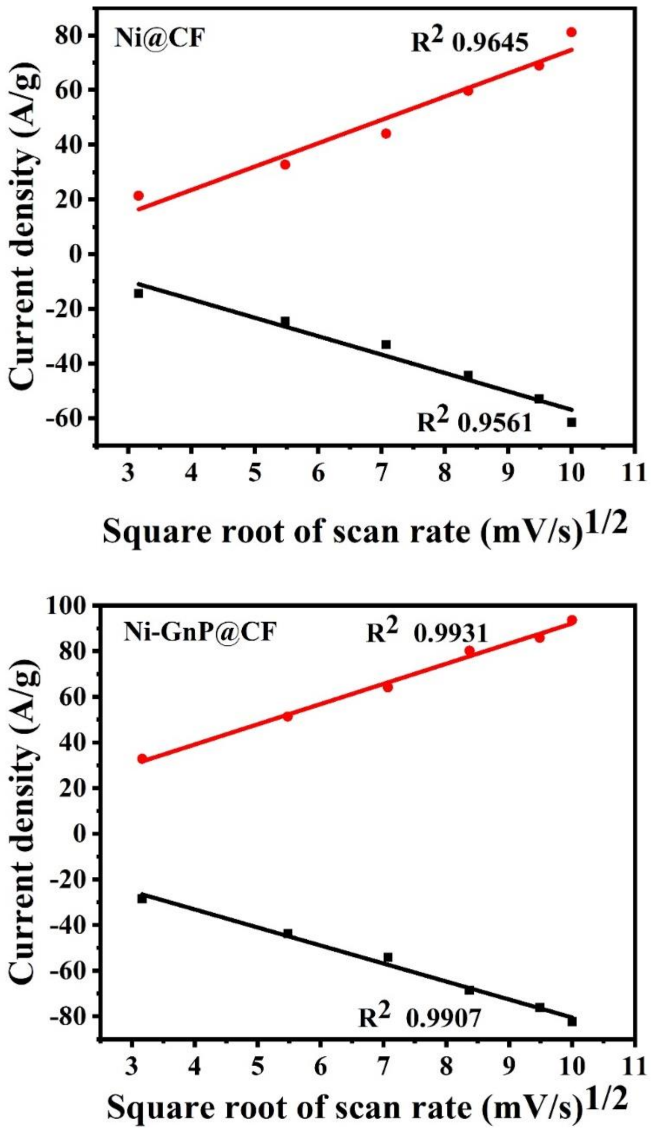

Figure S3. Plot for peak current density vs. square root of scan rates.

polymers

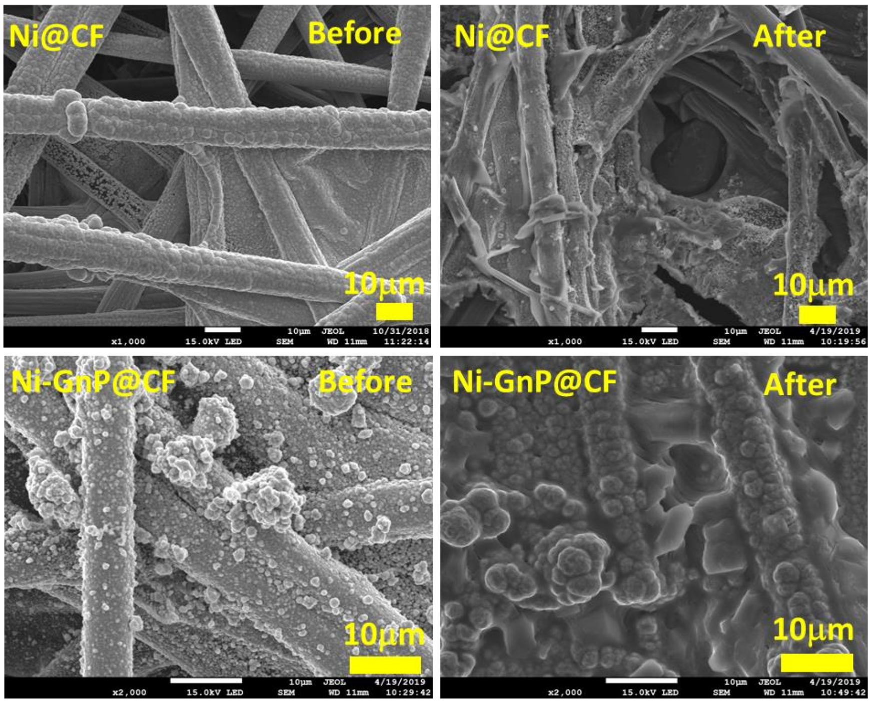

Figure S4. SEM comparison of both electrodes before and after 1000 cycles.
